# Experiences, Challenges and Informational Needs of Patients With Oral Epithelial Dysplasia

**DOI:** 10.1111/odi.15289

**Published:** 2025-02-17

**Authors:** Waleed Alamoudi, Richeal Ni Riordain, Stefano Fedele, Stephen Porter

**Affiliations:** ^1^ Faculty of Dentistry King Abdulaziz University Jeddah Saudi Arabia; ^2^ UCL Eastman Dental Institute University College London London UK; ^3^ Cork University Dental School and Hospital University College Cork Cork Ireland; ^4^ Biomedical Research Centre, NIHR University College London Hospitals London UK

**Keywords:** informational needs, oral dysplasia, oral precancer, patient experience, qualitative study

## Abstract

**Objectives:**

This study aims to explore the patients' experiences with oral epithelial dysplasia (OED) to identify associated clinical challenges and informational needs.

**Methods:**

Semi‐structured interviews, guided by a topic outline, addressed disease‐specific information, investigative procedures, treatments, impacts on quality of life, healthcare systems and information sources. The interviews were audio recorded, transcribed verbatim and analysed using thematic analysis.

**Results:**

A total of 30 individuals participated in the study. Four primary themes were identified: delays in diagnosis, knowledge about OED, the psychological impact of the disease and patient education.

**Conclusion:**

To our knowledge, this is the first qualitative study to explore the lived experiences of patients with OED. It highlights significant challenges, including accessing appropriate medical services, delays in diagnosis, physical and psychological burdens and the need for better education. Positive experiences were noted when patients received care from knowledgeable clinicians who provided consistent education and effective communication. The findings of this study may guide the future development of measurement tools on the outcome measures of individuals with OED.

## 1 Introduction

Oral epithelial dysplasia (OED) is a term used to describe various changes in the cells and structure of the oral epithelium associated with an increased likelihood of developing oral squamous cell carcinoma (OSCC) (Tilakaratne et al. 2019). OSCC ranks among the 15 most common types of cancer in the United Kingdom (UK), with over 6000 new cases identified annually (Cancer Research UK 2017). OED is estimated to affect 2.5–5 per 1000 individuals (Mehanna et al. 2009). Research has shown that OED can elevate the risk of OSCC by 6%–36%, depending on the degree of dysplastic changes (Field et al. 2015). Oral potentially malignant disorders can precede the development of OED (Kierce et al. 2021). These disorders include oral lichen planus (OLP), oral submucous fibrosis (OSF) and oral leukoplakia (OL). Regular surveillance and surgical removal are the recommended methods of treatment (Mehanna et al. 2009).

Achieving favourable long‐term health outcomes for patients with OED requires accurate diagnosis, optimal treatment options and a positive and satisfying healthcare experience (Doyle et al. 2013). Patient experience is multifaceted, encompassing various dimensions and perspectives. Definitions of patient experience can vary significantly among healthcare professionals and evolve, particularly in the dynamic healthcare sector (Wolf and Jason 2014). The Beryl Institute defines patient experience as ‘the sum of all interactions, shaped by an organisation's culture, that influence patient perceptions across the continuum of care’ (Wolf and Jason 2014). Core concepts of a positive patient experience include patient‐centred care, effective communication, patient education, patient and family partnerships, informational transparency and personalised and unique care (Wolf et al. 2021). Although satisfaction is essential to the overall patient experience, it is important to note that positive patient experiences are about much more than mere satisfaction. Satisfaction pertains to only certain periods in time, whereas the patient experience encompasses everything a patient encounters, the perspectives they carry with them and the narratives they share as a consequence (Wolf et al. 2021).

An obstacle that might arise during medical encounters is a disparity in the perception of complaints, signs or symptoms between the patient and the provider, resulting in inconsistencies in the approach to the disease and the strategy for management (Bensing 1991). To overcome this obstacle, qualitative research can offer insightful information about patients' subjective experiences and needs, thus facilitating more informed medical decision‐making and treatment approaches (Tong et al. 2016). Qualitative research is highly regarded as a good approach for examining important aspects of an individual's issues, such as pain, which may not be adequately explored using other research methods (Osborn and Rodham 2010).

It is crucial to highlight that patient experience extends beyond mere quantitative measurements and survey results, which typically offer insights into only specific stages or parts of an individual's path (Wolf et al. 2021). Therefore, to deliver detailed insights into everyday problems and human experiences, qualitative research examines phenomena within the contexts of individuals and groups (Moser and Korstjens 2017), offering a more versatile approach than quantitative research (Korstjens and Moser 2017). Previous studies on head and neck cancer (Scott et al. 2006; Deng et al. 2019) and chronic facial pain (Taimeh et al. 2023) have successfully utilised this method to investigate various aspects of patient experiences. Therefore, this study employed a qualitative approach using interviews. A thorough review of existing literature revealed a lack of research explicitly investigating the experiences of individuals with OED. Hence, this study aimed to provide a comprehensive understanding of the experiences, challenges and informational needs of patients with OED in a dental hospital in the UK.

## 2 Materials and Methods

### 2.1 Ethical Considerations and Study Registration

An impartial expert reviewed the study protocol and confirmed its rigour and feasibility. The study adhered to the Declaration of Helsinki guidelines for medical research involving human subjects. It was registered with the University College London Hospitals/University College London (UCLH/UCL) Joint Research Office under reference/EDGE number 153912 and IRAS project ID 318039. The study received a favourable opinion from the National Health Service (NHS) Research Ethics Committees, specifically the London–Surrey Borders Research Ethics Committee (reference 22/PR/1743). Additionally, it obtained ethical approval from the Health Research Authority and Health and Care Research Wales. Reporting of the qualitative component in this article complies with the guidelines outlined in the Standards for Reporting Qualitative Research (SRQR) checklist.

### 2.2 Study Design and Participants

This study was a semi‐structured, interview‐based, qualitative research project conducted at the UCLH Royal National ENT and Eastman Dental Hospitals' Oral Medicine Unit. Purposive sampling was used to select individuals diagnosed with OED through histopathological examination based on the 2017 World Health Organization classification system (El‐Naggar et al. 2017). The inclusion criteria for the study were adults aged 18 years or older, proficiency in both written and spoken English and the ability to provide informed consent. Eligible participants were recruited during their routine clinical visits. Qualitative sample size was determined by the principle of data saturation, which was reached at 30 participants, aligning with literature recommendations for semi‐structured interview studies of moderately heterogeneous patient groups (Sargeant 2012). The research team provided each participant with a detailed verbal explanation of the study's objectives and the expected outcomes of their involvement. Participants were then given an information sheet to review and were asked to sign an informed consent form.

### 2.3 Data Collection

Data collection occurred between March and December 2023 and continued until saturation was achieved. The saturation was defined as the point where no new emerging information would allow further development of a category's properties (Strauss 2017). Each interview lasted between 30 and 40 min, with an average duration of 35 min. All interviews were documented on paper and recorded in audio format. The interviews were conducted by two moderators (WA and RNR) who identified themselves as researchers and explicitly stated that they were not involved in the clinical service of any individuals. This precaution ensured that participants felt comfortable sharing adverse experiences without hesitation. The moderators, who had clinical backgrounds in oral medicine and were trained in qualitative research, took care to avoid influencing participants' responses with their ideas or opinions.

Participants provided data through semi‐structured interviews, which enabled the collection of open‐ended information while adhering to a set of guiding and predetermined questions (DeJonckheere and Vaughn 2019). A detailed topic guide was created for the interview discussion (Hancock et al. 2001), serving as a foundation for structured conversations and encouraging engagement between the researcher and participants. Key discussion topics covered a broad range of subjects, including initial appointments with primary healthcare providers, referrals to specialised healthcare facilities, progression to cancer risk, investigation procedures, treatment options, experiences with NHS services, information sources, and the physical and psychosocial impacts of OED. Participants were also free to bring up issues outside the framework that they deemed significant. Throughout the interviews, the guide was revised to obtain data that most effectively addressed the research objectives.

### 2.4 Data Analysis

Verbatim transcription was performed for all interviews. The researchers conducted a preliminary data assessment by engaging in reflective notetaking and forming initial impressions while listening to the audiotapes. Common themes within the responses of the participants were identified using thematic analysis. Through line‐by‐line coding, data were organised into subunits to facilitate pattern recognition. Codes with similar content were grouped to establish common categories. Recognising themes is a dynamic and interpretive task (Kiger and Varpio 2020). As a result, they were developed through an iterative inductive process, where coded data was merged, examined and interpreted. Each theme was subsequently accompanied by a detailed narrative description to provide context. Audit trails and data triangulation were applied to increase the reliability of the findings.

To determine data saturation, two researchers (WA and RNR) independently coded each set of three interviews before convening to compare emerging codes and subthemes. As coding progressed, earlier transcripts were revisited to ensure newly identified codes could be integrated. Saturation was deemed reached when no new codes or themes emerged over three consecutive interviews, indicating that further data collection would not deepen understanding of the topic (Strauss 2017).

## 3 Results

### 3.1 Participants

The study initially included a cohort of 35 participants, consisting of 24 females and 11 males. However, due to personal circumstances (*n* = 2) and time constraints (*n* = 3), only 30 participants consented to partake in the study, resulting in 21 females and nine males. Participants' ages ranged from 44 to 83 years, with a mean age of 64.4. The number of dysplastic sites varied between 1 and 7, averaging 1.8 per participant. The initial diagnosis of dysplasia occurred between 2 and 17 years before the start of the trial, averaging 7.3 years. The clinical features of the participants are presented comprehensively in Table 1.

**TABLE 1:** Clinical characteristics of participants.

Patient ID	Age (years)	Sex	Diagnosis (years)	Dysplasia sites	Location	Degree of dysplasia/associated disease
001	73	F	13	2	Buccal mucosa	Mild/OLP
002	44	M	12	1	Palate	Mild/OLP
003	63	F	17	1	Tongue	Mild/OLP
004	72	M	10	3	Buccal mucosa, palate	Moderate, severe/OLP
005	70	F	10	1	Tongue	Mild, moderate, severe/OLP
006	77	F	3	2	Floor of mouth, gingiva	Mild, moderate/OLP
007	68	F	6	2	Gingiva	Mild, moderate/OLP
008	79	F	4	7	Gingiva, palate	Moderate/OSCC
009	54	F	2	1	Tongue	Mild, moderate/OLP
010	66	F	6	1	Tongue	Moderate, severe/OSCC
011	63	M	3	1	Tongue	Moderate/OLP
012	44	F	7	1	Tongue	Mild, moderate, severe/OLP
013	55	M	11	5	Buccal mucosa, palate, gingiva	Mild, moderate/OSF, OSCC
014	65	F	4	3	Buccal mucosa, gingiva, floor of mouth	Mild, moderate/OLP
015	57	F	7	2	Buccal mucosa	Mild, moderate
016	50	M	16	2	Tongue, floor of mouth	Mild, moderate, severe/OLP
017	61	F	4	1	Tongue	Mild, moderate, severe/OLP
018	66	M	8	2	Buccal mucosa	Mild, moderate/OLP
019	58	M	3	1	Tongue	Mild/OLP
020	57	F	2	1	Buccal mucosa	Mild/OLP
021	67	M	2	1	Gingiva	Mild/OLP
022	58	F	2	1	Buccal mucosa	Moderate, severe/OLP
023	68	F	7	2	Palate, gingiva	Moderate, severe/OLP, OSCC
024	63	F	8	1	Tongue	Moderate/OLP
025	76	F	9	2	Buccal mucosa, gingiva	Mild, moderate, severe/OLP
026	70	F	6	1	Buccal mucosa	Mild/OLP
027	75	F	6	3	Buccal mucosa, tongue	Moderate, severe/OLP, OSF
028	57	F	3	1	Gingiva	Mild, moderate/OLP
029	68	F	11	1	Tongue	Mild, moderate/OLP
030	70	M	17	1	Buccal mucosa	Mild, moderate/OLP

Abbreviations: F, female; M, male; OL, oral leukoplakia; OLP, oral lichen planus; OSCC, oral squamous cell carcinoma; OSF, oral submucous fibrosis.

### 3.2 Themes

The interviews generated a variety of perspectives regarding experiences with OED. The participants' responses varied based on disease history and individual characteristics. Four primary themes emerged from the data analysis, which included (i) delays in OED diagnosis, (ii) knowledge about OED, (iii) psychological impact and (iv) patient education. Table 2 presents the themes and subthemes identified from the participants' responses, including some findings and supporting quotations.

**TABLE 2:** A complete spectrum of subthemes developed from the primary themes.

Theme	Subtheme	Supporting quotations
Delay in OED diagnosis	Patient's inability to identify abnormal signs and symptoms	‘Initially, it began as an ulcer in my cheek, and I assumed I just needed simple treatment in that area’ (001)
	Clinician incompetence	‘My GP referred me to an oral surgeon, suggesting that I should seek their expertise due to a potential issue with the skin in my mouth. I was sent back to my GP with no diagnosis; however, it was the oral medicine specialist who correctly identified and diagnosed the condition’ (012)
	Administrative issues	‘My referral was made incorrectly, necessitating a complete restart of the process. I was so frustrated’ (014)
Knowledge about OED	Nature of the disease	‘I believe that patients should be informed with all knowledge and utmost transparency about their diagnosis and disease’ (013)
	Aetiology and risk factors	‘I didn't know that alcohol can cause this in my mouth; I reduced the amount I drink and tried to stick to the recommended levels’ (011)
	Diagnostic tests and treatment options	‘It would be great to learn the particular aim of the biopsy sample and treatment alternatives’ (006)
Psychological impact	Diagnosis of OED	‘Upon receiving my initial diagnosis, I experienced a sense of worry, confusion, and disbelief, as there was a lack of awareness and understanding among others, and I did not encounter anyone who shared comparable experiences’ (005)
	Risk of progression to cancer	‘I'm extremely tired from the number of biopsies I've been having to chase any progression into cancer. It's draining and exhausting’ (025)
	Management adverse effects	‘You know, with mouth dryness, limited mouth opening, and graft I've got after the surgery, I'm not confident at a table—and that makes me sad’ (023)
Patient education	Regular education	‘I'd be grateful if the doctor would remind me of my plan each time I see him and not assume that I know everything I need to do because I only see him once a year and, as you can imagine. That's enough time for the information to fall through the cracks’ (026)
	Lack of reliable sources of information	‘Whenever I search for information, I exclusively rely on the NHS, as it provides a sense of security. However, I haven't found reliable sources for mouth precancer or dysplasia’ (013)
	Supplementary educational tools	‘As a non‐native English speaker, watching a video would be helpful to better understand the information’ (028)
	Group discussions	‘I'm interested in meeting other individuals who share the same issue in order to get insight from their experiences and compare them to my own. I propose establishing a recurring meeting to exchange experiences’ (014)

Abbreviation: OED, oral epithelial dysplasia.

#### 3.2.1 Delays in OED Diagnosis

Many participants expressed frustration about significant delays in their OED diagnosis, often attributed to the failure or inability to recognise abnormal signs and symptoms. Patients frequently perceived their symptoms as minor or temporary, which led them to ignore the issues, delay seeking medical care and a lack of urgency in addressing their condition.

For example, some participants reported:

Initially, it began as an ulcer in my cheek, and I assumed I just needed simple treatment in that area. (001)

I ignored it as I had ulcers as a child. I decided to wait it out and trust that it would resolve itself. I believed it was simply a mouth ulcer that eventually would go away.(009)

I wouldn't go to a doctor for a tiny discolouration under my tongue because they would think I'm exaggerating. (024)

Many individuals also expressed notable dissatisfaction with the competence of general practitioners (GPs) or other healthcare professionals, indicating a preference for the expertise of an OED specialist instead. In addition, this incompetence can lead to numerous clinical visits before receiving suitable medical attention was also reported. Many patients experienced a frustrating cycle of multiple hospital visits and referrals, often enduring considerable delays before being seen by an appropriate clinical team capable of addressing their healthcare needs effectively.

As one participant noted:

My GP referred me to an oral surgeon, suggesting that I should seek their expertise due to a potential issue with the skin in my mouth. I was sent back to my GP with no diagnosis; however, it was the OED specialist who correctly identified and diagnosed the condition. (012)

Another participant highlighted the complex nature of dysplasia symptoms:

One issue with dysplasia is that its symptoms can resemble those caused by other factors, such as lichen planus and certain medications. When I consulted a GP, the initial assumption was often the simplest explanation, as my GP immediately attributed my symptoms to menopause. Only the oral medicine specialist at this hospital recognised the true disease. (007)

Another reported the difficulty in securing a diagnosis:

I consulted with two general practitioners and one dentist; they all didn't know how to manage or where to refer me for the white patch I've had in my mouth for months. I ultimately ended up seeing a Maxfax surgeon who sampled the lesion and found out that dysplasia was evident. This whole journey took around two years to reach an accurate diagnosis—luckily, the lesion didn't progress into cancer. (021)

Additionally, many individuals faced significant administrative hurdles during the referral process, which led to prolonged and frustrating delays.

My referral was made incorrectly, necessitating a complete restart of the process. I was so frustrated. (014)

The referral protocols dealing with mouth dysplasia at this hospital or other hospitals have to be improved. (001)

I've done my research before seeking a referral, which was very difficult to get through. Without my investigation and persistence, I would not have arrived at this point. (019)

#### 3.2.2 Knowledge About OED

Several participants highlighted the critical importance of obtaining comprehensive knowledge about the diagnosis, nature of the disease, risk factors, treatment options and prognosis of OED. Before their encounters, none of the participants had any awareness of OED. Participants agreed that the moment of diagnosis marked a pivotal turning point, during which detailed information about all aspects of the condition should be communicated to ensure patients are fully informed and prepared to manage their health effectively.

As one participant expressed:

I believe that patients should be informed with all knowledge and utmost transparency about their diagnosis and disease.(013)

Another indicated the shock at learning about their condition:

I have never heard of it. I am familiar with breast and prostate cancer. I was surprised to learn that I had mouth precancer. (003)

One proposed the need for specialised patient‐specific service:

Is it possible to have a specialised mouth dysplasia clinic funded by the NHS? Specialists who possess comprehensive knowledge of the disease and its various manifestations and management? (004)

Many participants were unfamiliar with the aetiology and risk factors associated with OED. Several also lacked knowledge regarding the correlations connecting alcohol and HPV with OED.

One participant admitted:

I didn't know that alcohol can cause this in my mouth. I reduced the amount I drink and am trying to stick to the recommended levels. (011)

Another reported:

I know HPV can result in vaginal cancer, but in the mouth*—*never heard of that. (023)

The participants emphasised the importance of promptly receiving thorough information regarding routine diagnostic tests and available treatment options.

For example, some participants stated:

It would be great to learn the particular aim of the biopsy sample and treatment alternatives. (006)

Knowing that I may finally at least receive treatment for my issue was tremendously helpful to me.(015)

I was advised to have a surgical operation to remove my mouth lesions over regular watching. I appreciated the thorough knowledge I was given. (024)

#### 3.2.3 Psychological Impact

Several individuals reported that OED has affected their psychological well‐being. These impacts arise due to the diagnosis itself, the chronic nature of the condition, the uncertainty of progression to cancer and the treatments involved. Emotional distress was common at the first diagnosis, with feelings of worry and confusion due to lack of awareness.

One participant described:

Upon receiving my initial diagnosis, I experienced a sense of worry, confusion, and disbelief, as there was a lack of awareness and understanding among others, and I did not encounter anyone who shared comparable experiences.(005)

Another added:

Initially, everything was uncertain and ambiguous, as I lacked a clear understanding of what I had for many years. Hence, I was so stressed out and scared until I met Dr. xxx at this hospital. (008)

Upon discovering the meaning of oral dysplasia, the doctor informed me that it is a condition I must endure, as there is no solution available. He explained that the initial phases of the disease vary across individuals. I had significant distress due to my refusal to acknowledge it as a medical condition.(017)

Some participants expressed apprehension and anxiety about the potential progression to oral cancer and OED recurrence.

I'm extremely tired from the number of biopsies I've been having to chase any progression into cancer. It's draining and exhausting. (025)

If I had been aware of all the possibilities of having cancer when I received my diagnosis, I would have experienced greater peace of mind, as I have recently acquired a significant amount of knowledge. (006)

The risk, things that warrant cautionary attention. For instance, one of my colleagues was diagnosed with mouth cancer, which made me concerned about the possibility of developing a similar condition. Therefore, it is important to emphasise any relevant symptoms that may arise. If I were to experience any abnormal growth or hardness in that region, what course of action should I take? (029)

Participants also expressed challenges related to the management of OED, particularly the adverse effects that arose following major surgical procedures. Several individuals recognised the impact of these complications, including dry mouth, limited mouth opening, grafting and an inability to eat normally, on several aspects of their lives.

You know, with mouth dryness, limited mouth opening, and graft I've got after the surgery, I'm not confident at a table—and that makes me sad. (023)

When I look at myself in the mirror, my smile is not the same anymore, my confidence and intimacy with my husband have gotten affected. I had a couple of plastic surgeries to enhance the surgery's adverse effects, but that didn't really change a lot. (010)

The challenges I have in communication, particularly in my profession as a professor, have undeniably caused annoyance and impacted my life. (016)

#### 3.2.4 Patient Education

Participants highlighted the vital significance of receiving ample information and consistent education regarding OED. They conveyed satisfaction with the interactions they had with knowledgeable and skilled clinicians. There was a belief among patients that the provision of information about OED should be ongoing, as knowledge might change over time and relevant disease‐specific updates are difficult for non‐clinicians to find.

I'd be grateful if the doctor would remind me of my plan each time I see him and not assume that I know everything I need to do because I only see him once a year and, as you can imagine…that's enough time for the information to fall through the cracks. (026)

I've been having memory issues recently. I need to be reminded about the important information. (006)

I can't remember much about my disease because I had it a long time ago and never recurred. I always need to be reminded and educated. (018)

Several participants appreciated the support they received at diagnosis but thereafter felt abandoned due to a lack of reliable sources of information, which affected their acquisition of deeper knowledge about the condition. Consequently, they sought to gather information from other sources. They turned to the internet to gather information, which resulted in feelings of being swamped and discouraged.

One participant reported:

Whenever I search for information, I exclusively rely on the NHS, as it provides a sense of security. However, I haven't found reliable sources for mouth precancer or dysplasia. (013)

Others reported:

I believe it is beneficial to have a preliminary understanding, but upon initial diagnosis of any condition, one needs some time to fully comprehend and accept the situation, wouldn't you agree? It may be helpful to direct individuals to helplines or sources of additional information, such as online resources or support groups. (015)

If you access the internet or Google and encounter the issue of feeling sad due to observing an arbitrary, unskilled collective of individuals who engage in spreading scary narratives. (022)

Alongside individual clinical consultations, the participants emphasised their desire for more extensive information on OED. They cited a diverse array of supplementary educational resources, encompassing written materials such as printed documents and webpages, as well as audio‐visual content like YouTube videos. These supplementary tools would be beneficial for obtaining further comprehension of the information provided in the clinic and or to remind the patient of any forgotten information.

Some responses were:

As a non‐native English speaker, watching a video would be helpful to better understand the information. (028)

Videos could be easier to digest and understand. And yet, written information and wording is important, especially in advanced cases, as it reflects the seriousness and severity of the condition more than the videos. (002)

I can read the booklet anytime, while videos require an electronic device, which I can't afford. (006)

I prefer videos because of convenience. I can slow it down, repeat it, see pictures for better imagination. (017)

Some participants suggested attendance at group discussions. Through the exchange of experiences and advice, individuals had medical benefits. Furthermore, engagement with peers facilitated emotional and mental support.

I'm interested in meeting other individuals who share the same issue to get insight from their experiences and compare them to my own. I propose establishing a recurring meeting to exchange experiences. (14)

It would be beneficial to have the ability to share experiences, treatment alternatives, and outcomes with individuals who have comparable diagnoses. (022)

## 4 Discussion

This is the first qualitative study, to our knowledge, that investigates patient's experience with OED. The study identified four primary themes identified after data analysis: delays in diagnosis, knowledge about OED, psychological impact and patient education. The delayed diagnosis could be driven by patients' inability to recognise symptoms, clinician incompetence and administrative inefficiencies, often leading to lengthy referral processes. Participants also expressed a need for comprehensive knowledge upon diagnosis, including clarity on aetiology, risk factors, diagnostic tests, cancer development risks and treatments. The psychological impact was significant, with patients reporting uncertainty, confusion, worry and treatment‐related side effects that affected their quality of life. Additionally, participants highlighted gaps in patient education and support, emphasising the need for reliable resources, supplementary educational tools (e.g., pamphlets and videos) and group discussions to share experiences and coping strategies.

This study indicated that several factors may contribute to delays in diagnosis, including the inability of patients to identify abnormal signs and symptoms, clinician incompetence and healthcare administrative hurdles. Some patients reported not perceiving their symptoms as serious or indicative of premalignancy. This could be explained by the fact that early symptoms of OED are frequently subtle and painless, leading them to be mistaken for normal mouth issues and easily overlooked. This aligns with a study on advanced‐stage oral cancer (Rubright et al. 1996), where 87% of individuals reported being unable to identify warning signs during self‐examinations. However, the current analysis also indicates that experiencing concerning symptoms is not always essential for seeking quick aid, as some patients sought assistance shortly after noticing even mild symptoms, such as a change in colour. Patients who delayed seeking care expressed that they would have sought treatment earlier had they been aware of the seriousness of their symptoms.

The participants in this study also reported that some dentists and GPs demonstrated insufficient competence and training, particularly in assessing mucosal lesions in the mouth such as OED. According to several studies, primary care providers are hesitant to diagnose and manage this category of illnesses (Sardella et al. 2007; Bindakhil et al. 2021). The inability to perceive symptoms as indicative of something warranting serious attention by a clinician has been documented for testicular cancer (Gascoigne et al. 1999), breast cancer (Ramirez et al. 1999) and oral cancers (Scott et al. 2006; Gigliotti et al. 2019). However, distinguishing OED from other conditions such as OLP or OL can be challenging for non‐specialist clinicians due to overlapping clinical features. Findings indicate that many participants experienced diagnostic delays or uncertainties, a trend also noted in the literature (Sardella et al. 2007). This underscores the importance of improving the training of GPs and primary care teams in recognising subtle mucosal changes that may indicate dysplasia. These results highlight the value of targeted educational efforts and easily accessible resources for both patients and non‐specialist clinicians.

Our findings also show that participants were transferred repeatedly between several dentists and GPs, with these clinicians diagnosing the oral lesions incorrectly or not recognising the malignant potential and seriousness of the disease or because of a lack of knowledge about the appropriate centres for their complaints. This finding is consistent with a prior study, where individuals with chronic facial pain reported multiple referrals to both primary and secondary healthcare facilities in their attempts to get medical attention (Taimeh et al. 2023). Well‐coordinated referral pathways and stronger interprofessional collaboration could ensure timely management and boost patient confidence. Once participants in this study accessed specialist care, they reported clearer understanding and reduced anxiety. In addition, experts with varied experiences may employ different strategies for managing OED. For example, clinicians with an oral medicine background might suggest regular surveillance and non‐invasive treatments, whereas oral surgeons might favour surgical interventions (Mehanna et al. 2009). Therefore, a significant obstacle for patients with OED is a lack of established guidelines for referring patients and determining appropriate treatment techniques. Strengthening the standards of undergraduate and postgraduate training in this field could enhance the efficacy of achieving a timely diagnosis and appropriately managing OED.

The findings of this study demonstrated that participants' knowledge about OED was insufficient, particularly at the time of their initial diagnosis. This finding is consistent with previous research on oral cancer (de Amorim Póvoa et al. 2025). Additionally, this insufficiency can be attributed to several factors, including clinicians not providing enough information, complexity of information, rarity of OED may limit general awareness and long intervals between follow‐up appointments could lead to forgetfulness. However, once the diagnosis was established, the participants emphasised the importance of thorough and continued communication regarding essential disease‐related information. An earlier OED study highlighted that addressing this critical element can enhance shared decision‐making, mitigate the negative psychological impacts, improve future health outcomes and reduce healthcare expenditures (Alsoghier et al. 2022).

This study also showed that throughout the clinical course of the condition, the participants' levels of knowledge exhibited considerable variability. Some individuals demonstrated a high level of understanding about OED, often due to factors such as a long history of the disease, multiple recurrences with varying grades and a history of progression to cancer. Conversely, other patients in the current analysis displayed limited knowledge and understanding of OED, possibly attributed to factors such as a past diagnosis of a mild disease without progression or recurrence, older age or medical conditions affecting memory and comprehension. Some participants particularly emphasised the need for detailed information on the risk factors and potential progression to oral cancer, a need that has been corroborated by previous research on OED (Alsoghier et al. 2021). Furthermore, the results of this study underscore the importance of providing patients with comprehensive information about investigative tests and treatment options, aligning with findings from an earlier study on OED (Alsoghier et al. 2024).

Our findings show that several participants experienced significant psychological burdens from OED, adversely affecting their quality of life. These burdens were attributed to multiple factors, including delays in diagnosis, uncertainty about the disease, potential progression to cancer, risk of recurrence, challenges in controlling risk factors and management of adverse effects. A cross‐sectional study supports these findings, showing that patients with OED had lower quality‐of‐life scores (Ashshi et al. 2023). Another investigation revealed that patients with OED often experience heightened anxiety, fear and emotional distress due to concerns about the potential progression to mouth cancer (Alsoghier et al. 2021). The latter study found that 30% of participants elevated anxiety, 16% suffered from depression and 26% endured emotional distress. In addition to the adverse effects of investigative sampling and therapeutic surgical procedures involving tissue removal, the participants of this study experienced significant impairments in nutrition and speech. Other studies on OED confirm the negative impact of OED management on the quality of life (Alsoghier et al. 2021; Ashshi et al. 2023).

Of the 30 participants, those diagnosed with only mild dysplasia (001, 002, 003, 019, 020, 021, 026) underwent an incisional or punch biopsy followed by clinical observation, while the remainder underwent repeated surgical excisions over the years due to higher dysplasia grades, progression to cancer or recurrences. Although the overarching themes of diagnostic delays, psychological impact and the need for patient education were common to both groups, individuals who underwent major surgical procedures reported additional concerns regarding post‐operative complications (e.g., graft‐related difficulties, altered speech and dryness). Conversely, those on clinical surveillance pathways spoke more frequently of anxiety surrounding potential malignant transformation. These differences underscore the heterogeneity of patient experiences and highlight the importance of personalised approaches to patient support and education.

In the current study, the participants indicated that regular OED education is essential. The provision of continued education is a critical component in the clinical management of both malignancies (Ankem 2015) and premalignant conditions like OED (Alsoghier et al. 2024). Our findings also demonstrate that the primary and preferred source of information is direct, one‐on‐one meetings with an OED specialist. Indeed, verbal discussions remain the most effective and irreplaceable method of information exchange (Stewart 1995). The participants also expressed a desire to access additional information from other reliable resources, such as leaflets or videos. Evidence suggests that supplementary educational tools, including written materials (e.g., booklets) and audio‐visual aids (e.g., YouTube video clips), can enhance understanding and provide valuable support (Eckman et al. 2012).

Our analysis shows that, in the case of OED, which predominantly affects older individuals, written materials were favoured over videos by some patients due to factors like affordability and accessibility. Some participants also highlighted the importance of written information, particularly in advanced cases, as it conveys the gravity of the condition effectively. However, for non‐English speakers, videos were preferred as they offer visual aids to overcome language barriers. Additionally, support groups were noted to have a positive impact, providing both medical and emotional support through shared experiences and advice. This aligns with research indicating the beneficial role of support groups in aiding patients with cancer (Hoey et al. 2008).

### 4.1 Implications of This Study

This is the first qualitative study, to our knowledge, aimed at investigating the patient experience with OED. This study provides valuable insights into patient‐reported outcomes, enabling a better understanding of patient experiences (Rothman et al. 2009). Such findings can be helpful for the development and selection of instruments that effectively capture the lived experiences of individuals with OED. In addition, these findings also can be utilised to further inform a previously developed measurement tool for OED, the oral epithelial dysplasia informational needs questionnaire, created by Alsoghier et al. (2022). This approach ensures the content validity, sensitivity and responsiveness of measures and enhances their applicability in evaluating patient‐centred care for OED (Wiering et al. 2017).

### 4.2 Study Limitations

The study was conducted in specific dental department settings. Hence, the findings may lack generalisability to other populations or healthcare systems. In qualitative research, the researcher plays a pivotal role and can significantly shape the study's outcomes (Dodgson 2019). This underscores the concept of reflexivity, wherein researchers are aware of their impact on participants while acknowledging how the research process influences them personally (Gilgun 2008). Researchers must also guard against the Hawthorne effect, where participants may alter their behaviour due to awareness of being observed, potentially skewing results (Brinkman et al. 2007). Additionally, retrospective investigations may introduce errors in participant recollections, emphasising the need for caution. Given the exploratory nature of small‐sample studies, conducting larger‐scale research is vital to affirm findings and enhance the robustness of conclusions.

## Author Contributions

**Waleed Alamoudi:** conceptualization, methodology, data curation, investigation, formal analysis, project administration, writing – original draft, writing – review and editing. **Richeal Ni Riordain:** conceptualization, methodology, investigation, supervision, resources, writing – review and editing. **Stefano Fedele:** conceptualization, methodology, supervision, resources, writing – review and editing. **Stephen Porter:** conceptualization, methodology, supervision, project administration, resources, writing – review and editing.

## Conflicts of Interest

The authors declare no conflicts of interest.

## Data Availability

The data that support the findings of this study are available on request from the corresponding author. The data are not publicly available due to privacy or ethical restrictions.

## References

[odi15289-bib-0001] Alsoghier, A. , R. N. Riordain , S. Fedele , C. Liew , and S. Porter . 2022. “Information Needs and Oral Epithelial Dysplasia: Development and Psychometric Evaluation of a Novel Instrument.” Oral Diseases 28, no. 1: 76–86. 10.1111/odi.13726. 33200486

[odi15289-bib-0002] Alsoghier, A. , R. N. Riordain , S. Fedele , and S. Porter . 2021. “Psychosocial Impacts of Oral Epithelial Dysplasia.” Journal of Oral Pathology & Medicine 50, no. 7: 700–707. 10.1111/jop.13173. 33728714

[odi15289-bib-0003] Alsoghier, A. , R. N. Riordain , S. Fedele , and S. Porter . 2024. “Patient and Clinician Perspectives of Information Needs Concerning Oral Epithelial Dysplasia.” Oral Diseases 30, no. 4: 2166–2175. 10.1111/odi.14668. 37455497

[odi15289-bib-0004] Ankem, K. 2015. “Assessing Cancer Patients' Health Information Needs: A Standardized Approach.” Information Research 20, no. 2. https://InformationR.net/ir/20‐2/paper668.html.

[odi15289-bib-0005] Ashshi, R. A. , D. Stanbouly , P. G. Maisano , et al. 2023. “Quality of Life in Patients With Oral Potentially Malignant Disorders: Oral Lichen Planus and Oral Epithelial Dysplasia.” Oral Surgery, Oral Medicine, Oral Pathology, Oral Radiology 135, no. 3: 363–371. 10.1016/j.oooo.2022.11.006. 36549944

[odi15289-bib-0006] Bensing, J. 1991. “Doctor‐Patient Communication and the Quality of Care.” Social Science & Medicine 32, no. 11: 1301–1310. 10.1016/0277-9536(91)90047-g. 2068614

[odi15289-bib-0007] Bindakhil, M. , S. Charmelo‐Silva , A. A. Bin Dakhil , and I. A. ALOmair . 2021. “The Value of the Oral Medicine Specialty in the Modern Healthcare Systems.” Saudi Journal of Health Systems Research 1, no. 2: 33–40.

[odi15289-bib-0008] Brinkman, W. B. , S. R. Geraghty , B. P. Lanphear , et al. 2007. “Effect of Multisource Feedback on Resident Communication Skills and Professionalism: A Randomized Controlled Trial.” Archives of Pediatrics & Adolescent Medicine 161, no. 1: 44–49. 10.1001/archpedi.161.1.44. 17199066

[odi15289-bib-0009] CANCER RESEARCH UK . 2017. “Oral Cancer Incidence Statistics.” December 10, 2024. https://www.cancerresearchuk.org/health‐professional/cancer‐statistics/statistics‐by‐cancer‐type/oral‐cancer/incidence.

[odi15289-bib-0010] DeJonckheere, M. , and L. M. Vaughn . 2019. “Semistructured Interviewing in Primary Care Research: A Balance of Relationship and Rigour.” Family Medicine and Community Health 7, no. 2: e000057. 10.1136/fmch-2018-000057. 32148704 PMC6910737

[odi15289-bib-0011] Deng, J. , R. J. Sinard , and B. Murphy . 2019. “Patient Experience of Head and Neck Lymphedema Therapy: A Qualitative Study.” Support Care Cancer 27, no. 5: 1811–1823. 10.1007/s00520-018-4428-2. 30167789

[odi15289-bib-0012] Dodgson, J. E. 2019. “Reflexivity in Qualitative Research.” Journal of Human Lactation 35, no. 2: 220–222. 10.1177/0890334419830990. 30849272

[odi15289-bib-0013] Doyle, C. , L. Lennox , and D. Bell . 2013. “A Systematic Review of Evidence on the Links Between Patient Experience and Clinical Safety and Effectiveness.” BMJ Open 3, no. 1: e001570. 10.1136/bmjopen-2012-001570. PMC3549241 23293244

[odi15289-bib-0014] Eckman, M. H. , R. Wise , A. C. Leonard , et al. 2012. “Impact of Health Literacy on Outcomes and Effectiveness of an Educational Intervention in Patients With Chronic Diseases.” Patient Education and Counseling 87, no. 2: 143–151. 10.1016/j.pec.2011.07.020. 21925823

[odi15289-bib-0015] El‐Naggar, A. K. , J. K. C. Chan , J. R. Grandis , T. Takata , and P. J. Slootweg . 2017. WHO Classification of Head and Neck Tumours. 4th ed. International Agency for Research on Cancer (IARC).

[odi15289-bib-0016] Field, E. A. , C. E. McCarthy , M. W. Ho , et al. 2015. “The Management of Oral Epithelial Dysplasia: The Liverpool Algorithm.” Oral Oncology 51, no. 10: 883–887. 10.1016/j.oraloncology.2015.06.015. 26198978

[odi15289-bib-0017] Gascoigne, P. , M. D. Mason , and E. Roberts . 1999. “1999. Factors Affecting Presentation and Delay in Patients With Testicular Cancer: Results of a Qualitative Study.” Psycho‐Oncology 8, no. 2: 144–154. 10.1002/(SICI)1099-1611(199903/04)8:2<144::AID-PON349>3.0.CO;2-P. 10335558

[odi15289-bib-0018] Gigliotti, J. , S. Madathil , and N. Makhoul . 2019. “Delays in Oral Cavity Cancer.” International Journal of Oral and Maxillofacial Surgery 48, no. 9: 1131–1137. 10.1016/j.ijom.2019.02.015. 30878273

[odi15289-bib-0019] Gilgun, J. F. 2008. “Lived Experience, Reflexivity, and Research on Perpetrators of Interpersonal Violence.” Qualitative Social Work 7, no. 2: 181–197.

[odi15289-bib-0020] Hancock, B. , E. Ockleford , and K. Windridge . 2001. An Introduction to Qualitative Research. Trent focus group.

[odi15289-bib-0021] Hoey, L. M. , S. C. Ieropoli , V. M. White , and M. Jefford . 2008. “Systematic Review of Peer‐Support Programs for People With Cancer.” Patient Education and Counseling 70, no. 3: 315–337. 10.1016/j.pec.2007.11.016. 18191527

[odi15289-bib-0022] Kierce, J. , Y. Shi , H. Klieb , N. Blanas , W. Xu , and M. Magalhaes . 2021. “Identification of Specific Clinical Risk Factors Associated With the Malignant Transformation of Oral Epithelial Dysplasia.” Head & Neck 43, no. 11: 3552–3561. 10.1002/hed.26851. 34472151

[odi15289-bib-0023] Kiger, M. E. , and L. Varpio . 2020. “Thematic Analysis of Qualitative Data: AMEE Guide No. 131.” Medical Teacher 42, no. 8: 846–854. 10.1080/0142159X.2020.1755030. 32356468

[odi15289-bib-0024] Korstjens, I. , and A. Moser . 2017. “Series: Practical Guidance to Qualitative Research. Part 2: Context, Research Questions and Designs.” European Journal of General Practice 23, no. 1: 274–279. 10.1080/13814788.2017.1375090. 29185826 PMC8816399

[odi15289-bib-0025] Mehanna, H. M. , T. Rattay , J. Smith , and C. C. McConkey . 2009. “Treatment and Follow‐Up of Oral Dysplasia—A Systematic Review and Meta‐Analysis.” Head & Neck 31, no. 12: 1600–1609. 10.1002/hed.21131. 19455705

[odi15289-bib-0026] Moser, A. , and I. Korstjens . 2017. “Series: Practical Guidance to Qualitative Research. Part 1: Introduction.” European Journal of General Practice 23, no. 1: 271–273. 10.1080/13814788.2017.1375093. 29185831 PMC8816396

[odi15289-bib-0027] Osborn, M. , and K. Rodham . 2010. “Insights Into Pain: A Review of Qualitative Research.” Reviews in Pain 4, no. 1: 2–7. 10.1177/204946371000400102. PMC4590060 26527327

[odi15289-bib-0028] Póvoa, L. S. D. A. , D. A. Souza , F. S. Verner , R. B. Junqueira , and S. N. de Aquino . 2025. “Lack of Knowledge, Understanding, and Delayed Attitudes Towards Health‐Seeking Behavior in Oral Cancer Patients: A Qualitative Pilot Study.” Oral Surgery, Oral Medicine, Oral Pathology, Oral Radiology 139, no. 1: 64–72. 10.1016/j.oooo.2024.08.024. 39462707

[odi15289-bib-0029] Ramirez, A. J. , A. M. Westcombe , C. C. Burgess , S. Sutton , P. Littlejohns , and M. A. Richards . 1999. “Factors Predicting Delayed Presentation of Symptomatic Breast Cancer: A Systematic Review.” Lancet 353, no. 9159: 1127–1131. 10.1016/s0140-6736(99)02142-x. 10209975

[odi15289-bib-0030] Rothman, M. , L. Burke , P. Erickson , N. K. Leidy , D. L. Patrick , and C. D. Petrie . 2009. “Use of Existing Patient‐Reported Outcome (PRO) Instruments and Their Modification: The ISPOR Good Research Practices for Evaluating and Documenting Content Validity for the Use of Existing Instruments and Their Modification PRO Task Force Report.” Value in Health 12, no. 8: 1075–1083. 10.1111/j.1524-4733.2009.00603.x. 19804437

[odi15289-bib-0031] Rubright, W. C. , H. T. Hoffman , C. F. Lynch , et al. 1996. “Risk Factors for Advanced‐Stage Oral Cavity Cancer.” Archives of Otolaryngology – Head & Neck Surgery 122, no. 6: 621–626. 10.1001/archotol.1996.01890180029009. 8639293

[odi15289-bib-0032] Sardella, A. , F. Demarosi , G. Lodi , L. Canegallo , L. Rimondini , and A. Carrassi . 2007. “Accuracy of Referrals to a Specialist Oral Medicine Unit by General Medical and Dental Practitioners and the Educational Implications.” Journal of Dental Education 71, no. 4: 487–491. 17468309

[odi15289-bib-0033] Sargeant, J. 2012. “Qualitative Research Part II: Participants, Analysis, and Quality Assurance.” Journal of Graduate Medical Education 4, no. 1: 1–3. 10.4300/JGME-D-11-00307.1. 23451297 PMC3312514

[odi15289-bib-0034] Scott, S. E. , E. A. Grunfeld , J. Main , and M. McGurk . 2006. “Patient Delay in Oral Cancer: A Qualitative Study of Patients' Experiences.” Psychooncology 15, no. 6: 474–485. 10.1002/pon.976. 16142843

[odi15289-bib-0035] Stewart, M. A. 1995. “Effective Physician‐Patient Communication and Health Outcomes: A Review.” CMAJ 152, no. 9: 1423–1433. 7728691 PMC1337906

[odi15289-bib-0036] Strauss, A. L. 2017. The Discovery of Grounded Theory: Strategies for Qualitative Research. Routledge.

[odi15289-bib-0037] Taimeh, D. , R. N. Riordain , S. Fedele , and R. Leeson . 2023. “Healthcare Priorities in Patients With Chronic Facial Pain of Temporomandibular Disorders.” Oral Diseases 29, no. 7: 2878–2887. 10.1111/odi.14483. 36565449

[odi15289-bib-0038] Tilakaratne, W. M. , P. R. Jayasooriya , N. S. Jayasuriya , and R. K. De Silva . 2019. “Oral Epithelial Dysplasia: Causes, Quantification, Prognosis, and Management Challenges.” Periodontology 2000 80, no. 1: 126–147. 10.1111/prd.12259. 31090138

[odi15289-bib-0039] Tong, A. , R. L. Morton , and A. C. Webster . 2016. “How Qualitative Research Informs Clinical and Policy Decision Making in Transplantation: A Review.” Transplantation 100, no. 9: 1997–2005. 10.1097/TP.0000000000001358. 27479165

[odi15289-bib-0040] Wiering, B. , D. de Boer , and D. Delnoij . 2017. “Patient Involvement in the Development of Patient‐Reported Outcome Measures: The Developers' Perspective.” BMC Health Services Research 17, no. 1: 635. 10.1186/s12913-017-2582-8. 28886742 PMC5591531

[odi15289-bib-0041] Wolf, C. P. X. P. , and A. Jason . 2014. “Defining Patient Experience.” Patient Experience Journal 1, no. 1: 7–19.

[odi15289-bib-0042] Wolf, J. A. , V. Niederhauser , D. Marshburn , and S. L. LaVela . 2021. “Reexamining “Defining Patient Experience”: The Human Experience in Healthcare.” Patient Experience Journal 8, no. 1: 16–29.

